# *Avicennia germinans* (black mangrove) vessel architecture is linked to chilling and salinity tolerance in the Gulf of Mexico

**DOI:** 10.3389/fpls.2014.00503

**Published:** 2014-09-26

**Authors:** Eric N. Madrid, Anna R. Armitage, Jorge López-Portillo

**Affiliations:** ^1^Department of Marine Biology, Texas A&M University at GalvestonGalveston, TX, USA; ^2^Red de Ecología Funcional, Instituto de Ecología, A.C.Xalapa, México

**Keywords:** elasticity, evolution, phenotypic plasticity, safe xylem hydraulic architecture, salt marsh, *Spartina alterniflora*, vessel architecture, wetland

## Abstract

Over the last several decades, the distribution of the black mangrove *Avicennia germinans* in the Gulf of Mexico has expanded, in part because it can survive the occasional freeze events and high soil salinities characteristic of the area. Vessel architecture may influence mangrove chilling and salinity tolerance. We surveyed populations of *A. germinans* throughout the Gulf to determine if vessel architecture was linked to field environmental conditions. We measured vessel density, hydraulically weighted vessel diameter, potential conductance capacity, and maximum tensile fracture stress. At each sampling site we recorded mangrove canopy height and soil salinity, and determined average minimum winter temperature from archived weather records. At a subset of sites, we measured carbon fixation rates using a LI-COR 6400XT Portable Photosynthesis System. Populations of *A*. *germinans* from cooler areas (Texas and Louisiana) had narrower vessels, likely reducing the risk of freeze-induced embolisms but also decreasing water conductance capacity. Vessels were also narrower in regions with high soil salinity, including Texas, USA and tidal flats in Veracruz, Mexico. Vessel density did not consistently vary with temperature or soil salinity. In abiotically stressful areas, *A. germinans* had a safe hydraulic architecture with narrower vessels that may increase local survival. This safe architecture appears to come at a substantial physiological cost in terms of reduction in conductance capacity and carbon fixation potential, likely contributing to lower canopy heights. The current distribution of *A. germinans* in the Gulf is influenced by the complex interplay between temperature, salinity, and vessel architecture. Given the plasticity of *A. germinans* vessel characters, it is likely that this mangrove species will be able to adapt to a wide range of potential future environmental conditions, and continue its expansion in the Gulf of Mexico in response to near-term climate change.

## INTRODUCTION

Coastal wetland environments in the tropics are primarily comprised of mangrove forests, but salt marshes are dominant in temperate latitudes (Saintilan et al., 2014). In the northern half of the western hemisphere, there is a transition between these two ecosystems in the Gulf of Mexico along the roughly 2200 km stretch of shoreline between La Pesca, Tamaulipas (Mexico) and Tampa, FL (USA). Over the last several decades, populations of mangroves (e.g., *Avicennia* spp.) have begun to expand into areas of salt marsh (*Spartina alterniflora* and other species) along the Texas, Louisiana, and Florida coasts, as well as in Australia and other regions of the world (Perry and Mendelssohn, 2009; Everitt et al., 2010; Krauss et al., 2011; Montagna et al., 2011; Williamson et al., 2011; Raabe et al., 2012; Bianchi et al., 2013; Cavanaugh et al., 2014). In some regions, forests of *Avicennia germinans* have completely replaced *S*. *alterniflora* (Sherrod and McMillan, 1981). The distribution of *A*. *germinans* is expected to continue expanding over the next several decades in response to climate change, which may alter the frequency of freezing temperatures, the amount of rainfall, and the rate of sea level rise (Osland et al., 2013; Cavanaugh et al., 2014).

Many species of salt marsh plants can survive in both temperate and tropical climates, but mangrove forests are found exclusively in tropical and subtropical latitudes (Tomlinson, 1994; Duke et al., 1998; Spalding et al., 2010). *Avicennia germinans* is the only mangrove tree species found in the subtropical northern Gulf of Mexico. Thus, the anatomical and physiological adaptations of *A*. *germinans* may play an important role in determining the range of mangrove forest distribution, and consequently of salt marsh distribution, in the northern Gulf of Mexico, and will influence the potential for further mangrove expansion in the region. Given the important global role of mangroves as ecosystem engineers, this expansion may have implications for a variety of ecosystem services (Ellison et al., 2005).

Temperature is widely recognized as a major driver of these coastal changes (e.g., McMillan and Sherrod, 1986; Osland et al., 2013; Cavanaugh et al., 2014). Interactions between biotic and abiotic characteristics of mangrove trees and their surrounding environment are also likely to influence the rate of change. For example, freezing temperatures may be fatal to mangroves, especially in arid and saline soils, contributing to low soil water potential (Quisthoudt et al., 2012); this creates more negative xylem pressures, increasing the likelihood of catastrophic embolism formation (Stuart et al., 2007). Embolisms are pockets of air that can form following cavitation, when sudden changes in hydraulic tension (e.g., due to a rapid drop in temperature or water stress in drought conditions) in the xylem cause liquid water to vaporize. After a period of time, air will fill these vapor-filled conduits, forming embolisms that disrupt the transport of xylem sap (Tyree and Sperry, 1989). Embolisms can sometimes be repaired when pressure within the xylem forces air out of the conduit through pits (pores) in the lateral sides of the vessels and back into solution (Holbrook and Zwieniecki, 1999), though in severe cases, embolism formation can cause plant mortality (Urli et al., 2013). Therefore, plants with better resistance to embolism are more likely to survive conditions, such as freezing temperatures, that cause rapid changes in xylem pressure. Laboratory studies have demonstrated that several mangrove tree species with narrower vessel elements are better able to prevent the formation of freeze-induced embolisms and consequently have a greater chilling tolerance (Stuart et al., 2007), though vessel plasticity and chilling tolerance vary widely among mangrove species (Chave et al., 2009).

Based on previous laboratory work on mangroves (Stuart et al., 2007), we expected that in field populations, *A. germinans* xylem vessel architecture (using hydraulically weighted vessel diameter and density as proxies) would vary with winter temperature minima and soil salinity. Hydraulically weighted vessel diameter (Davis et al., 1999; Stuart et al., 2007; Charrier et al., 2013) is a relatively unexplored trait in any mangrove species across a large spatial scale. Therefore, our objective was to investigate how xylem vessel characteristics influence mangrove distribution by systematically comparing *A. germinans* vessel anatomy across its distribution in the Gulf of Mexico. We hypothesized that vessel architecture would vary across the Gulf of Mexico, with narrower vessels and greater vessel densities in more northern latitudes.

## MATERIALS AND METHODS

**FIGURE 1:**
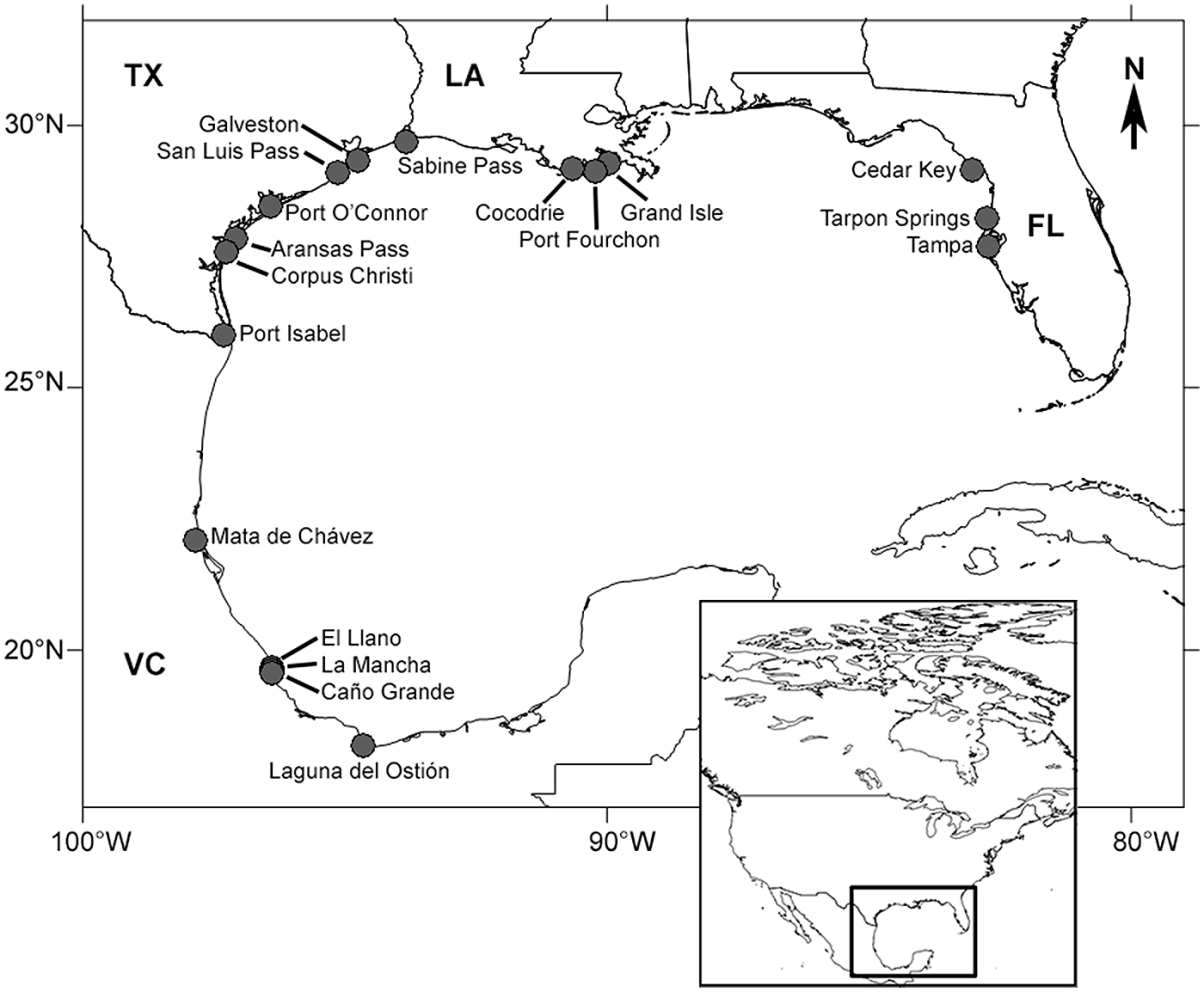
**Mangrove collection sites in the Gulf of Mexico.** Sites were located in Florida (FL, USA), Louisiana (LA, USA), Texas (TX, USA), and Veracruz (VC, Mexico).

Field sites were coastal or estuarine *A. germinans* (black mangrove) populations distributed throughout the Gulf of Mexico. Sites were grouped into four regions of the Gulf: Veracruz (Mexico), and Texas, Louisiana, and FL (USA; **Table 1**; **Figure 1**). Each region was considered to be a separate population for statistical analyses. Average winter temperature minima were determined from climatic records (previous 10–30 years) archived in the National Oceanic and Atmospheric Administration’s National Climatic Data Center (**Table 1**). Entire pieces of stem (1–1.5 cm thick and ≈30 cm long) were clipped from three or more mature (reproductive) *A. germinans* trees at each site (one per tree, *n* ≥ 3). Within each site, all sampled trees were approximately the same height and at least 10 m apart. At the USA sites, samples were collected from *A. germinans* stands in the mid- to high intertidal zone. In Veracruz, wood samples were collected from two distinct zones. In high salinity mudflat or sand flat areas, termed “tidal flats,” *A. germinans* typically had dwarf morphology (<3 m tall). In low to moderate salinity interdistributary flood basins, trees were typically over 10 m tall. The Veracruz tidal flat and flood basin localities were treated as separate populations in statistical analyses. Samples were collected at chest height for taller trees or at a height of about one meter for shorter trees. We chose stems with fresh, healthy, green leaves. Each sample was placed in a sealed plastic bag, transported to the laboratory, and dried at 60°C for 7–10 days.

**Table 1:** Site locations and average minimum winter (December-February) temperatures over a 10–30 year period.

Site	Region	Latitude	Longitude	Average winter minimum temperature (°C)
Tampa	Florida	27° 41.957′N	82° 42.885′W	18.0
Tarpon Springs	Florida	28° 13.136′N	82° 45.249′W	17.5
Cedar Key	Florida	29° 9.123′N	83° 1.841′W	14.2
Grand Isle	Louisiana	29° 15.796′N	89° 58.212′W	15.3
Port Fourchon	Louisiana	29° 6.929′N	90° 12.822′W	15.3
Cocodrie	Louisiana	29° 10.480′N	90° 38.925′W	15.3
Sabine Pass	Texas	29° 41.332′N	93° 50.576′W	15.4
Galveston	Texas	29° 19.935′N	94° 44.890′W	18.5
San Luis Pass	Texas	29° 5.125′N	95° 8.376′W	18.5
Port O’Connor	Texas	28° 27.813′N	96° 24.982′W	17.9
Aransas Pass	Texas	27° 51.110′N	97° 5.049′W	19.6
Corpus Christi	Texas	27° 35.677′N	97° 16.414′W	19.6
Port Isabel	Texas	26° 0.448′N	97° 18.464′W	19.5
Mata de Chávez	Veracruz: Tidal flat	22° 5.508′N	97° 51.652′W	21.2
El Llano	Veracruz: Tidal flat	19° 39.947′N	96° 24.173′W	21.2
La Mancha	Veracruz: Tidal flat	19° 35.588′N	96° 23.217′W	21.2
Ca no Grande	Veracruz: Flood basin	19° 33.883′N	96° 23.262′W	21.2
Laguna del Ostión	Veracruz: Flood basin	18° 10.710′N	94° 38.280′W	21.2

*Average winter temperature minima were determined from climactic records (10–30 years) archived in the National Oceanic and Atmospheric Administration’s National Climatic Data Center.*

The transverse edge of each wood sample presented a cross section of the mangrove stem. This edge was ground with progressively finer grades of sandpaper (200, 400, 800 grit) and cut into sections 0.5–1.0 mm thick. Each section was dehydrated through a graded ethanol series, immersed in Histo-Clear (National Diagnostics, Atlanta, GA, USA), placed on a slide with the sanded side facing upward, and mounted to a slide and coverslip with Permount mounting medium (Fisher Scientific, Fair Lawn, NJ, USA). Slides were viewed on an Axiophot microscope (Carl Zeiss Microscopy, LLC, Thornwood, NY, USA) at 200x magnification with differential interference contrast optics. Three equally spaced areas of 8.12 × 10^-2^ mm^2^ in each wood section were photographed with an Axiophot camera, and xylem characteristics were calculated with Zeiss Axiocam software. The radial growth of *Avicennia* is relatively unique among mangroves in that it occurs through several vascular cambia that are arranged in patches, creating non-concentric groups of xylem tissue surrounded by internal phloem tissue (Robert et al., 2014). The cross sections we examined were large enough to capture the heterogeneity of this complex patchiness (Robert et al., 2011). We focused our examinations on the outermost portion of the stems, which contained the youngest xylem and were most likely to be related to the environmental conditions we measured in the soil samples. Hydraulically weighted vessel diameter (hereafter “vessel diameter”) was calculated as: D_h_ = ∑ D^5^/∑ D^4^ where D was the diameter of each individual xylem vessel (Sobrado, 2007; Charrier et al., 2013). Total cross-sectional vessel area (sum of the cross-sectional areas of all xylem vessels per field of view, scaled to mm^2^ of stem) was calculated as a relative measure of potential conductance capacity (Davis et al., 1999). Vessel density was calculated as the number of xylem vessels per mm^2^ of stem, not excluding internal phloem from the stem area measurements.

One soil core (2 cm wide, 10 cm deep) was taken at the base of every sampled tree. The section of the core between 2.5 and 5.0 cm from the surface of the soil was removed, placed in a sealed bag, and transported to the laboratory for further analysis. In the laboratory, each soil sample was dried at 70°C for at least 72 h. Soil salinity was measured by filtering a portion of a saturated soil paste (Richards, 1954) through two layers of No. 2 Whatman filter paper onto a Leica® temperature-compensated refractometer. The saturated soil paste technique was preferred over measurements of pore water salinity because salinities in tidally influenced brackish and salt marshes frequently change and soil paste-derived measurements of soil salinity provide a better long-term indication of relative salinity history across large distances than single “snapshot” measurements of porewater salinity.

Mangrove canopy height was calculated around trees where wood samples were collected (*n* ≥ 3/site). A digital photograph was taken parallel to the ground at a height of ∼1.5 m; each photograph contained a benchmark of known height (e.g., a one meter tall marker pole). The pixel height of the benchmark was determined. Pixel length was converted to meters and used as a coarse but consistent calculation of canopy height.

Biomechanics tests were performed on three or more wood samples per site after sections had been prepared for vessel diameter measurements. Each sample was dried for several days before analysis in order to desiccate the xylem parenchyma. Bark was peeled away to expose the xylem before each fracture test. In each test, a wood sample (standardized to 25 cm long) was placed in an MTS Insight 30 kN (kilonewton) tensile tester (MTS Systems Corporation, Eden Prairie, MN, USA) configured to conduct a three-point flexural test. The sample supports had a span of 20 cm and the head moved at a speed of 0.03 cm/s. Maximum tensile fracture stress (hereafter “maximum stress;” N/mm^2^) at fracture was calculated as: S_max_ = 3PL/πR^3^ where S_max_ was the maximum stress at fracture, P was the maximum load (lbf) at fracture, L was the support span (mm), and R (mm) was the average radius of the sample at its center and each edge.

Photosynthetic performance was measured on mangroves at two sites in Texas: Galveston and Sabine Pass (**Figure 1**). Real-time photosynthetic measurements and fluorescence light curves were collected on cloud- and rain-free days between May 5 and May 20, 2011. Preliminary observations indicated that rates of carbon fixation in *A. germinans* were positive between 0900 and 1300, but all measurements for this study were collected between 0900 and 1100 to avoid mid-day photosynthetic depression. Data were collected with a LI-6400XT Portable Photosynthesis System (LI-COR, Lincoln, NE, USA) following Madrid et al. (2012). In brief, carbon fixation, as estimated by chlorophyll fluorescence, was measured simultaneously with oxygen consumption at 1800, 1500, 1000, 500, 250, 100, 50, 25, 15, and 0 μmol m^-2^ s^-1^ PAR on five mature trees/site (one tree/day). Oxygen consumption potential and carbon fixation rates were compared with previously published values in order to assess the relative photosynthetic performance of *A. germinans* in the northern Gulf of Mexico.

To reduce the heteroscedasticity of variances, all dependent variables (canopy height, soil salinity, hydraulically weighted vessel diameter, vessel density, total vessel area, and maximum stress) were square root transformed and normalized to a mean of zero and a standard deviation of one. Data were analyzed with one-way Analysis of Variance (ANOVA), with region (Florida, Louisiana, Texas, Veracruz flood basin, and Veracruz tidal flat) as the independent variable. Differences among regions were assessed with *post hoc* Tukey tests. Principal components analysis (PCA) was used to explore which variables contributed to differences among regions, and to assess relationships among the dependent variables.

## RESULTS

**FIGURE 2:**
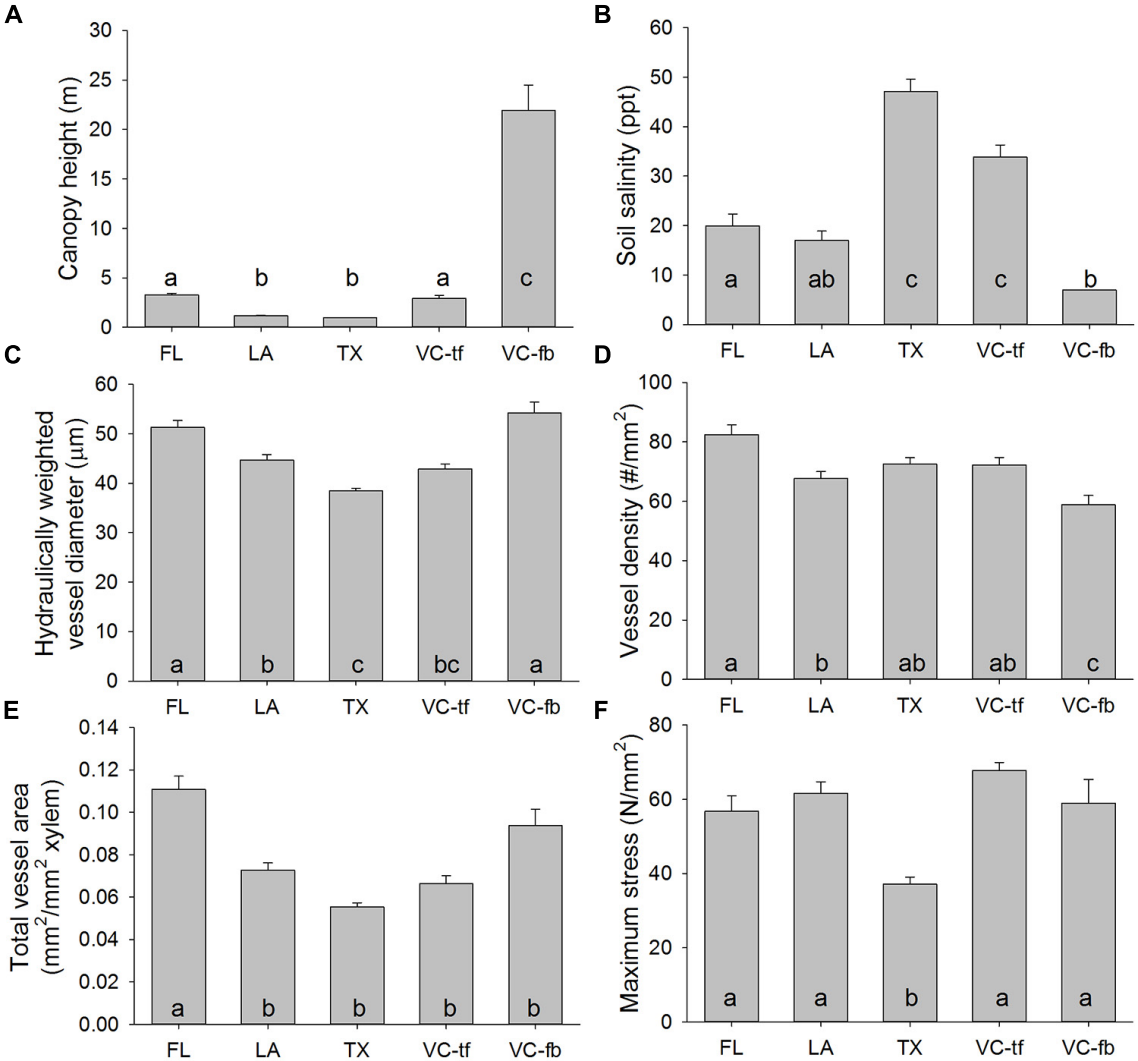
**Anatomical, biomechanical, soil, and canopy features of *Avicennia germinans* throughout the Gulf of Mexico: Florida (FL), Louisiana (LA), Texas (TX), Veracruz tidal flats (VC-tf), and Veracruz interdistributary flood basin (VC-fb). (A)** Mangrove canopy height; **(B)** Soil salinity; **(C)** Hydraulically weighted xylem vessel diameter; **(D)** Xylem vessel density; **(E)** Total xylem vessel area; **(F)** Maximum tensile fracture stress of mangrove branches. Error bars represent one SE. Letters indicate significant differences among regions from Tukey *post hoc* tests.

Mangrove canopy height varied significantly among study regions (**Table 2**). The tallest trees exceeded 20 m in height and were found in the southernmost interdistributary flood basin sites in Veracruz, Mexico (**Figure 2A**). In contrast, trees in Texas and Louisiana were often less than one meter tall. Trees in Florida and the Veracruz tidal flat areas were intermediate in height, about three meters tall.

**Table 2:** Results of one-way ANOVA comparing tree and environmental characteristics among five regions of the Gulf of Mexico.

	df	MS	*F*	*P*
Canopy height	4	40.0	270.2	<0.001
Soil salinity	4	19.4	33.6	<0.001
Hydraulically weighted vessel diameter	4	19.5	32.8	<0.001
Vessel density	4	4.6	4.9	0.001
Total vessel area	4	11.9	15.6	<0.001
Maximum stress	4	12.2	19.8	<0.001

Soil salinity varied significantly among regions (**Table 2**). Soil salinity exceeded 30 parts per thousand (ppt) at sites in Texas and the Veracruz tidal flat areas, but was less than 10 ppt in the Veracruz interdistributary flood basins (**Figure 2B**).

Vessel characteristics varied significantly among regions (**Table 2**), but not all characteristics had the same spatial patterns. Hydraulically weighted vessel diameters were lowest in Texas and up to 30% wider in Florida and the Veracruz flood basins (**Figure 2C**). Vessel density was relatively similar among sites, though it was higher in Florida than in Louisiana or the Veracruz flood basins (**Figure 2D**). Total cross-sectional vessel area was up to 50% higher in Florida than in all other study regions (**Figure 2E**). Maximum stress at fracture was more than 50% lower in Texas mangroves than at all other sites (**Figure 2F**; **Table 2**).

**FIGURE 3:**
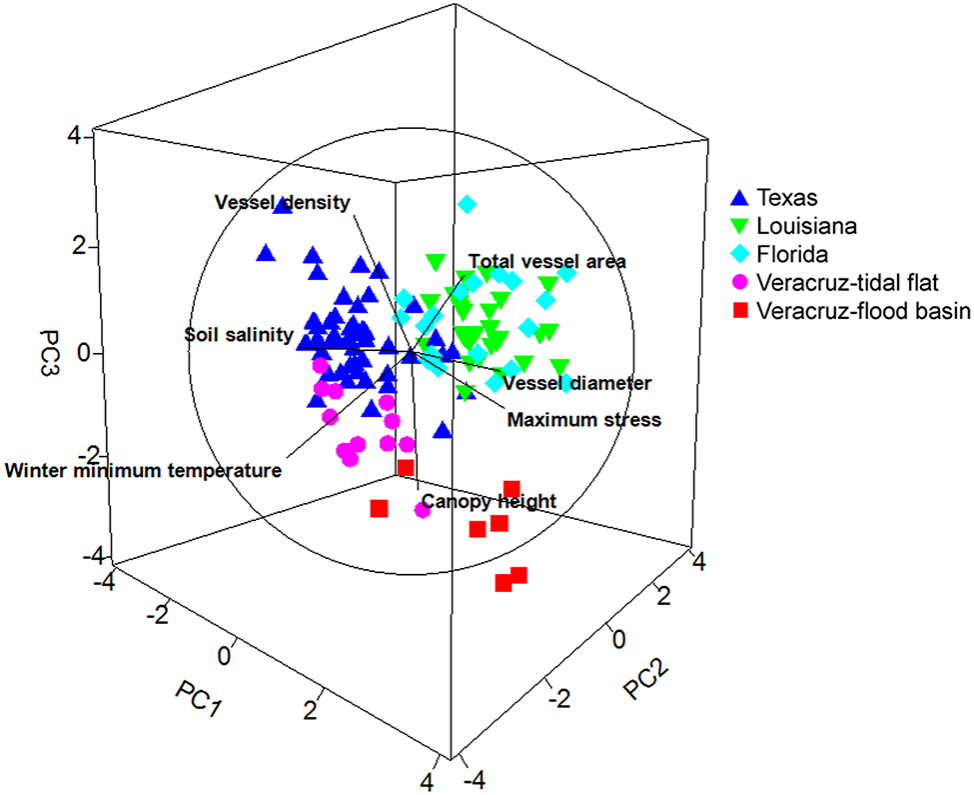
**Principal components analysis depicting the three significant principal components and the relative relationships among study regions and among plant and environmental characters.** Units follow **Figure 2**.

Principal components analysis revealed three significant principal components (PC; eigenvalue >1) that accounted for 76.8% of the variability among samples. The first PC (34.8%) was positively correlated (coefficient ≥0.35) with vessel diameter, total vessel area, and maximum stress at fracture, and inversely correlated with soil salinity (**Table 3**; **Figure 3**). The second PC (22.9%) was inversely correlated with vessel density, soil salinity, canopy height, and minimum winter temperature. The third PC (19.1%) was positively correlated with canopy height and total vessel area, and inversely correlated with canopy height and minimum winter temperature.

**Table 3:** Summary of results from principal components analysis, showing the correlation coefficients of each response variable with the three significant principal components.

	PC1	PC2	PC3
Canopy height (cm)	0.341	-**0.358**	-**0.516**
Soil salinity	-**0.406**	-**0.349**	0.017
Hydraulically weighted vessel diameter (μm)	**0.564**	-0.106	-0.037
Vessel density (#/mm^2^)	0.059	-**0.432**	**0.594**
Total vessel area (mm^2^/mm^2^ xylem)	**0.512**	-0.286	**0.348**
Maximum stress (n/mm^2^)	0.318	0.314	-0.277
Minimum temperature	-0.184	-**0.610**	-**0.427**
Total variance explained (%)	34.8	22.9	19.1

*Strong correlations (≥0.35) are indicated in bold type.*

All five regions had distinct tree characteristics and environmental conditions (**Figure 3**). Trees in Florida were generally aligned with PC1, indicating that they tended to grow in moderate salinity soils and had wider hydraulically weighted vessel diameters and higher total vessel area (**Table 3**; **Figure 2**). Trees in Louisiana were correlated with PC2 and PC3, indicating that they grew in a cooler climate and had shorter canopies and narrower vessels. Trees in Texas were inversely related to PC1, indicating that they grew in higher soil salinities and had narrower vessels, lower vessel area, and shorter canopies (**Table 3**; **Figure 2**). Trees in the Veracruz flood basins were directly related to PC1 and inversely related to PC3, suggesting that they grew in a warmer climate with lower salinity soils and had taller canopies, wider vessels, lower vessel density, and higher total vessel area. Trees in the Veracruz tidal flat areas were inversely related to PC3, suggesting that they had lower vessel density, lower total vessel area, a relatively tall canopy, and grew in a warmer climate.

**FIGURE 4:**
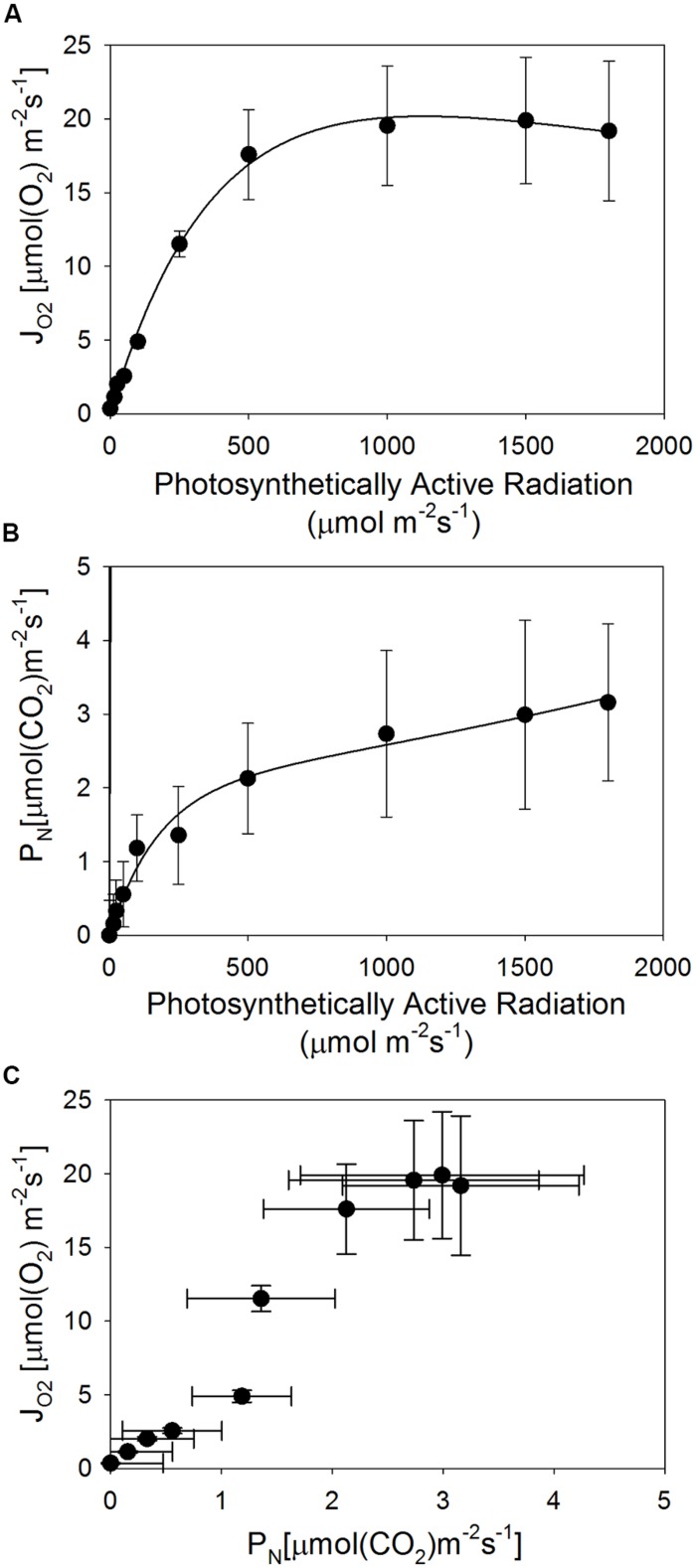
**Average photosynthetic performance of *A. germinans* across two sites in Texas. (A)** Oxygen consumption potential (J_O2_); **(B)** carbon fixation rates (P_N_); **(C)** the relationship between oxygen consumption potential and carbon fixation rates, as a measure of overall photosynthetic efficiency. Error bars represent one SD.

*Avicennia germinans* photochemical oxygen consumption (J_O2_) potential at two Texas sites neared maximum at 500 PAR (**Figure 4A**). Rates of carbon fixation (P_N_) were less than those of oxygen consumption, and were still increasing at the maximum PAR used in this study (1800; **Figure 4B**). To assess overall photosynthetic efficiency, the rates of oxygen consumption and carbon fixation were compared. The relationship between J_O2_ and P_N_ was non-linear (**Figure 4C**), indicating inefficient carbon fixation.

## DISCUSSION

Populations of *A*. *germinans* from cooler areas, namely those populations in Texas and Louisiana, had narrower vessels than populations from warmer habitats, particularly those in Florida and the Mexico fringe zones. At relatively high hydraulic tension, wider vessels will cavitate more than narrower vessels – as air bubbles come out of solution during the freezing and thawing process, they coalesce more easily in wider vessels, thus causing embolisms (Davis et al., 1999). Therefore, narrower vessels can reduce the frequency of freeze-induced embolisms and potentially increase chilling tolerance (Stuart et al., 2007; Charrier et al., 2013). Our study confirmed the link between vessel diameter and freezing risk in the field. Vessel characteristics in trees are often plastic (Chave et al., 2009), sometimes responding to environmental conditions on seasonal time scales (Arnold and Mauseth, 1999) and influencing cavitation risk in response to environmental stressors including freezing temperatures and drought (Choat et al., 2012). Recent physiological studies in *Avicennia marina* demonstrate that patchy xylem growth in this clade has allowed for constant and purposeful changes in wood anatomy in response to abiotic factors including salinity and drought (Robert et al., 2011, 2014). Our lab methodology sought to capture the variability introduced by xylem patchiness within the stem, and sampling a large number of trees across many study sites made our dataset relatively robust for drawing regional-level conclusions. Therefore, our study provides convincing field evidence that *A. germinans* may use plastic vessel architecture to tolerate the cooler subtropical climate of the northern Gulf of Mexico.

Our findings also provide an explanation for the results observed by McMillan (1975), Markley et al. (1982), and McMillan and Sherrod (1986) in their transplant experiments. These authors collected *A. germinans* from some of the localities where we collected our samples (Port Isabel, Aransas Pass, Galveston, Port Fourchon, Cedar Key, Tampa) and demonstrated through freezing and transplant experiments that plants from Texas had greater freeze tolerance than those from Louisiana, and that plants from Louisiana had greater freeze tolerance than those from Florida. Accordingly, we found that vessels were narrower in Texas than in Louisiana, and narrower in Louisiana than in Florida. Therefore, it is likely that the different *A. germinans* freezing tolerances among regions (McMillan, 1975; Markley et al., 1982; McMillan and Sherrod, 1986) are at least partially driven by differences in vessel diameters.

Although hydraulically weighted vessel diameter was linked to local temperature minima, it was also related to other environmental factors, namely soil salinity. In fact, our analysis revealed that soil salinity was more strongly correlated with the first two principal components, suggesting that vessel characteristics may be more strongly linked to soil salinity than to winter temperature. Vessels were narrowest in the regions with higher soil salinity, including Texas and Veracruz tidal flats. The Veracruz tidal flat was among the warmer sites, with no freezing risk. Therefore, the small vessel diameter in that region was likely driven by the extremely high soil salinity. This inverse relationship between salinity and vessel diameter had not been previously reported in *A. germinans*, though other mangrove species, including *Laguncularia racemosa* (Sobrado, 2007) and *A. marina* (Robert et al., 2009) have narrower vessels in saline conditions.

In contrast to the link between soil salinity and vessel diameter, we did not observe a strong relationship between vessel density and soil salinity. In species from warmer climates where freezing events are less likely, mangroves often have higher vessel densities at higher salinities (Schmitz et al., 2006; Sobrado, 2007; Robert et al., 2009). In these cases, vessel density increases at high salinity as a form of “safe hydraulic architecture,” where vessel redundancy helps protect the plant from the effects of cavitation, thus preserving the capacity of the water transport system (Robert et al., 2009). In the case of high salinity, a portion of the vessels may fail due to freeze- or salinity-induced embolism, but if vessel density is high, then there are likely to be other vessels to compensate for the loss, and hydraulic conduction capacity is maintained (Schmitz et al., 2006; Ewers et al., 2007). In our case study, *A. germinans* formed narrower vessels that are less susceptible to embolism formation in response to cooler temperatures, but did not produce them at an increased density. Consequently, as soil salinity increases and temperature goes down, water conduction capacity in *A*. *germinans* will decrease.

Lower water conductance capacity is likely linked to a decrease in productivity. Accordingly, we observed very low rates of carbon fixation in mangrove populations with high soil salinity and low temperature minima. The carbon fixation potentials we measured in *A. germinans* from Galveston and Sabine Pass, Texas were near 3 μmol CO_2_ m^-2^ s^-1^. In contrast, previous studies documented rates of carbon fixation (reported in μmol CO_2_ m^-2^ s^-1^) from 10 to 15 in populations of *A*. *germinans* (Krauss et al., 2006), 15 in *A. marina* (Naidoo, 2006) and 10 in *L. racemosa* (Sobrado, 2005) from the tropics. Carbon fixation is the primary source of energy derived through photochemistry, and efficient fixation would be indicated by a linear relationship between J_O2_ and P_N_ with a slope of one. Instead, we detected a non-linear relationship between J_O2_ and P_N_ and values of P_N_ were much less than J_O2_. Thus, energy transfer to the carbon fixation reactions was inefficient, lowering the overall rates of carbon fixation. This is a typical water stress response (Chaves et al., 2009) and is likely due to the extremely low water potentials of the highly saline soils in which the plants were growing, the reduced diameter and relatively low density of their vessels that result in low water transport capacities, or both.

Mangrove canopy height is frequently linked to temperature, with shorter canopies at higher latitudes (Méndez-Alonzo et al., 2008). This geographic pattern is often directly attributed to tree dieback in freeze-prone areas (Stevens et al., 2006; Soares et al., 2012). Our study demonstrated that vessel architecture also played a role in limiting canopy height. In particular, *A. germinans* in cooler areas had developed a safe hydraulic architecture with a higher density of narrower vessels, and these vessel features also occurred in high salinity areas. Although these vessel characteristics are likely effective at increasing survival in abiotically stressful areas, they have substantial physiological costs in terms of reduction in xylem conductivity and carbon fixation potential. Therefore, areas with frequent freezing events or extremely saline soils will require vessels with such a narrow diameter that photosynthetic output may not be sufficient for long-term growth, reproduction, and survival. Thus, while *A*. *germinans* vessel architecture is conducive to survival in cooler climates, there is a limit to this capacity. The populations we surveyed in the northwestern Gulf of Mexico likely have the most extreme vessel sizes and density and are in the most stressful abiotic conditions that *A*. *germinans* is capable of tolerating.

The current distribution of *A. germinans* in the Gulf of Mexico is likely limited by the complex interplay between temperature, hydroperiod, and salinity, and vessel architecture. However, environmental conditions in the Gulf are expected to change in response to near-term climate change. Likely outcomes include increases in temperature (Keim et al., 2011), thus decreasing freezing risk for mangroves. Decreases in precipitation and increased urban and agricultural demand for freshwater are likely to increase estuarine salinity (Gibson et al., 2005; Keim et al., 2011), possibly increasing salinity stress. Several models predict that changes in temperature, salinity, and sea level will cause mangrove expansion into higher latitudes (Doyle et al., 2010; Osland et al., 2013). This expansion has already been documented in many regions, including the Gulf of Mexico (e.g., Saintilan et al., 2014). Given the wide variation range of *A. germinans* vessel characters, it is likely that this mangrove species will be able to thrive in a wide range of potential future environmental conditions, and continue its near-term expansion at the expense of salt marshes in the Gulf of Mexico.

## Conflict of Interest Statement

The authors declare that the research was conducted in the absence of any commercial or financial relationships that could be construed as a potential conflict of interest.
